# HDMAC: A Web-Based Interactive Program for High-Dimensional Analysis of Molecular Alterations in Cancer

**DOI:** 10.1038/s41598-020-60791-z

**Published:** 2020-03-03

**Authors:** Chung Chang, Chan-Yu Sung, Han Hsiao, Jiabin Chen, I.-Hsuan Chen, Wei-Ting Kuo, Lung-Feng Cheng, Praveen Kumar Korla, Ming-Jhe Chung, Pei-Jhen Wu, Chia-Cheng Yu, Jim Jinn-Chyuan Sheu

**Affiliations:** 1 Department of Applied Mathematics, National Sun Yat-sen University, Kaohsiung, Taiwan, ROC; 2 Institute of Biomedical Sciences, National Sun Yat-sen University, Kaohsiung, Taiwan, ROC; 3 Division of Transplant Surgery/Urology, Department of Surgery, Kaohsiung Veterans General Hospital, Kaohsiung, 81362 Taiwan, ROC; 4 Department of Pharmacy, College of Pharmacy and Health Care, Tajen University, Pingtung County, 90741 Taiwan, ROC; 5 School of Medicine, National Yang-Ming University, Taipei, 112 Taiwan, ROC; 6 Division of Transplant Surgery/Urology, Department of Surgery, Tri-Service General Hospital, National Defense Medical Center, Taipei, 114 Taiwan, ROC; 7 Department of Biotechnology, Kaohsiung Medical University, Kaohsiung, 80708 Taiwan, ROC; 8 School of Chinese Medicine, China Medical University, Taichung, 40402 Taiwan, ROC; 9 Department of Health and Nutrition Biotechnology, Asia University, Taichung, 41354 Taiwan, ROC

**Keywords:** Genome informatics, Medical research, Oncology

## Abstract

Recent advances in high-throughput genomic technologies have nurtured a growing demand for statistical tools to facilitate identification of molecular changes as potential prognostic biomarkers or drugable targets for personalized precision medicine. In this study, we developed a web-based interactive and user-friendly platform for high-dimensional analysis of molecular alterations in cancer (HDMAC) (https://ripsung26.shinyapps.io/rshiny/). On HDMAC, several penalized regression models that are suitable for high-dimensional data analysis, Ridge, Lasso and adaptive Lasso, are offered, with Cox regression for survival and logistic regression for binary outcomes. Choice of a first-step screening is provided to address the multiple-comparison issue that often arises with large-volume genomic data. Hazard ratio or estimated coefficient is provided with each selected gene so that a multivariate regression model may be built based on the genes selected. Cross validation is provided as the method to estimate the prediction power of each regression model. In addition, R codes are also provided to facilitate download of whole sets of molecular variables from TCGA. In this study, illustration of the use of HDMAC was made through a set of data on gene mutations and a set on mRNA expression from ovarian cancer patients and a set on mRNA expression from bladder cancer patient. From the analysis of each set of data, a list of candidate genes was obtained that might be associated with mutations or abnormal expression of genes in ovarian and bladder cancers. HDMAC offers a solution for rigorous and validation analysis of high-dimensional genomic data.

## Introduction

Recent advances in high-throughput technologies such as microarrays and next generation sequencing have enabled researchers to identify molecular changes that are associated with cancers in a systematic way^1,2^. Such efforts have attracted much attention as the molecular changes may represent potential prognostic biomarkers or drugable targets for personalized precision medicine. Meanwhile, several multiple-data platforms, *e.g*., the Cancer Genome Atlas (TCGA) and Genotype-Tissue Expression (GTEx), have also become available to researchers when identifying genome-wide molecular changes of individual cancers^3,4^. With these updated tools and consortiums, there emerges a growing demand for statistical tools to facilitate identification of molecular changes.

There are several web tools available for researchers to analyze genomic data. For example, cBioPortal provides simultaneous display of RNA expression, mutations, copy number alterations and protein expression with multiple choices of plots for visualization^5,6^. HPA and Protein Expression Atlas are specialized in protein expression. The former is good at integrating protein information and the latter provides multi-species expression data^7,8^. There are also tools that provide analysis on specific molecules such as miRGator for miRNAs^9^. As useful as all these tools are, researchers always have specific demands in their studies that cannot be well addressed by the existing platforms. For example, with deepening understanding of cancer-associated genetic alterations, it becomes imperative to explore whether the changes are associated with clinical variables and survival and binary outcomes, and how. A few preliminary attempts have been made to generate new platforms to meet specific needs of researchers^10–12^, but a platform that is capable of handling high dimensional data is still lacking.

Genomic data are usually high dimensional, often with information of thousands of gene loci obtained from a much smaller number of patients, say, hundreds, and an even smaller number of clinical parameters. When the number of genes is larger than the number of subjects, standard regression models that are commonly used in statistical analysis become overwhelmed. Penalized regression models, such as the ridge regression, the least absolute shrinkage and selection operator (Lasso) regression, and the adaptive Lasso regression, provide attractive alternatives^13–17^. These methods typically result in shrinkage of the size of the regression coefficients. Specifically, the ridge regression reduces the magnitude of the coefficients while the Lasso and the adaptive Lasso force some of the coefficients to become zero. In addition, the Lasso regression estimator is sparse, *i.e*., many components are exactly 0 and Lasso automatically deletes unnecessary covariates, and the adaptive Lasso estimator is even sparser. Thus both the Lasso and the adaptive Lasso can be used for variable selection, with the latter selecting fewer variables than the former. In fact, these penalized regression methods have been widely used in large-scale genetic studies in recent years, such as identification of gene-gene interactions, gene selection in a high-dimensional cancer classification problem and a transcriptome analysis of pancreatic cancer survival^18–20^. Unfortunately, although these methods are heavily used in genetic analysis, they have not been incorporated in user-friendly web-based programs.

Aside from the high-dimensionality, the multiple-testing problem also needs to be addressed. In genomic studies, typically a test statistic and its corresponding p-value between one gene and the outcome variable are calculated to measure the extent of the association between them. When many tests are conducted at the same time, a lot of false positives (false discoveries) may arise. In fact, the false discovery rate (FDR) has become a key concept in recent large-scale genetic studies^21^. Unfortunately, such a function is rarely offered in currently available web tools and apps^22–24^. Therefore, proper statistical algorithms are thus needed to address the FDR issue.

Therefore, we aimed to develop a web-based interactive and user-friendly platform to fulfill the following goals. First, it would fit the regression models with survival and binary outcomes and high-dimensional genetic covariates, with the option of including clinical covariates. It would also identify important genetic alterations and construct a fitted multivariate regression model based on the identified genes. Further, it would choose a penalty type for the corresponding penalized regression model for high-dimensional data. It would offer a choice of a first-step screening to screen out unrelated variables if the multiple-testing problem is of concern. Last but not the least, it would estimate the prediction power for each regression model using cross validation with the correct p-values for the Lasso and adaptive Lasso provided. We also aimed to provide all relevant codes on GitHub for users’ convenience.

## Materials and Methods

The platform was written and all statistical analysis was performed with the statistical computing and graphic drawing language, R, with the help of Shiny, an R package that facilitates the building of interactive web Apps straight from R^25,26^.

### Clinical data

The data for developing the platform and the associated statistical analysis were downloaded from TCGA. It is also possible to download TCGA data from cBioPortal, but only limited numbers of genetic entries may be downloaded each time. We therefore wrote R codes to download large numbers of genomic data from TCGA, and the codes are available at GitHub (https://github.com/chung-R/HD-MAC). It is worth noting that users can use our App to run any available genetic datasets while TCGA is just an important source.

Two sets of data were obtained for this study. One contained 316 patients with serous type high-grade ovarian cancer, the most common and malignant form of ovarian cancer. The data contained detected mutations in 8,310 genes and expression information of 18,263 expressed mRNA entries, as well as the patients’ clinical parameters including age, stage, overall survival, disease-free survival and lymphovascular invasion. The other set of data included 189 patients with bladder cancer. It had expression information of 18,335 expressed mRNA entries and the patients’ clinical parameters including age, sex, stage, tumor invasion type, disease-free survival and overall survival. The Z score data of mRNA expression were used to indicate the deviation from the mean of each gene’s expression level. A Z score above 2 or below −2 was considered abnormal. In addition, to search for major genetic events in cancer-driving genes with minimal statistical bias, a preliminary screening was performed so that only the genes whose mutations were found in 1% or more and the mRNA entries whose abnormal expression was found in 2% or more of the patients were included. As a result, 670 mutated genes and 9,548 expressed mRNA entries of ovarian cancer and 8,024 expressed mRNA entries of bladder cancer were included in the final analysis below.

### Statistical methods

#### Ridge, lasso and adaptive lasso logistic regression

To identify genetic alterations associated with binary clinical outcomes, logistic regression based methods were used.

For logistic regression, the data are (*x*_*i*_, *y*_*i*_), *i* = 1, …, *n*, where *x*_*i*_ = (*x*_*i*1_, …, *x*_*iM*_) is the covariate of the *i*th subject such as copy number variation (CNV), gene expression and mutation (M is the number of genes) and *y*_*i*_ is the binary response for the *i*th subject such as stage (advanced stage vs. early stage) and tumor subtype (invasive vs. non-invasive).

The logistic regression model may be written as follows:

$$\mathrm{ln}(\frac{{p}_{i}}{1-{p}_{i}})=\frac{\exp ({x}_{i}\beta )}{1+\exp ({x}_{i}\beta )},$$

where *p*_*i*_ = P(*y*_*i*_ = 1*x*_*i*_) and β = (*β*_*i*_, …, *β*_*M*_)^T^ is the regression coefficient vector. Let *L*(β) be the log-likelihood for this model.

To address the high-dimensionality of the genomic data, we considered three regularized logistic regression models, ridge logistic regression, Lasso logistic regression and adaptive Lasso logistic regression^13,14^. The ridge logistic regression estimator ${\hat{\beta }}^{r}$^15^ can be obtained by minimizing

$$-L({\rm{\beta }})+{\lambda }_{\gamma }\mathop{\sum }\limits_{j=1}^{M}{\beta }_{j}^{2},$$

The Lasso logistic regression estimator ${\hat{\beta }}^{l}$^27^ can be obtained by minimizing

$$-L(\beta )+{\lambda }_{l}\mathop{\sum }\limits_{j=1}^{M}|{\beta }_{j}|.$$

The adaptive Lasso logistic regression estimator ${\hat{\beta }}^{al}$ can be obtained by minimizing

$$-L(\beta )+{\lambda }_{al}\mathop{\sum }\limits_{j=1}^{M}\frac{1}{{w}_{j}}|{\beta }_{j}|,$$

where *w*_*j*_ = ${\hat{\beta }}_{j}^{l}$ is the jth component of ${\hat{\beta }}^{l}$.

We used the cross-validation method to get the optimal tuning parameter estimators, ${\hat{\lambda }}_{r}$, ${\hat{\lambda }}_{l}$ and ${\hat{\lambda }}_{al}$. Then the genes selected by the Lasso and adaptive Lasso regression were evaluated based on their association with the binary outcome variable, invasive vs. non-invasive bladder cancer here.

#### Ridge, lasso and adaptive lasso cox models

To associate genetic alterations with the survival outcome, the Cox proportional hazards (PH) model was used.

The survival data are (*Z*_*j*_, *δ*_*i*_, *x*_*i*_) where *Z*_*i*_, *δ*_*i*_ and *x*_*i*_ are the observed time, right censoring indicator and the high-dimensional genetic covariates (such as CNV, gene expression and mutation) of the *i*th subject, respectively. *Z*_*i*_ = *min*(*T*_*i*_, *C*_*i*_), where *T*_*i*_ and *C*_*i*_ are the failure time and the right censoring time of the *i*th subject, respectively. *δ*_*i*_ = 1 if *T*_*i*_ < *C*_*i*_ and *δ*_*i*_ = 0 if *T*_*i*_ > *C*_*i*_. Assume *T*_*i*_ and *C*_*i*_ are independent conditional on *x*_*i*_. Here *T*_*i*_ is the disease-free survival time or overall survival time.

Similar to the above, three regularized Cox PH models, ridge, Lasso and adaptive Lasso, were used to analyze the survival data with high-dimensional covariates^16,17^. The hazard function given *x*_*i*_ in the Cox PH model is defined as follows:

$$h(t|{x}_{i})=\,{h}_{0}(t){e}^{{x}_{i}\alpha },$$

where $a={({\alpha }_{1},\ldots ,{\alpha }_{M})}^{T}$ is the regression coefficient vector.

Let *PL*(*α*) be the log partial likelihood for the Cox PH model. The Cox ridge regression estimator ${\hat{\alpha }}^{r}$ can be obtained by minimizing

$$-PL(\alpha )+{\lambda }_{r}^{PH}\mathop{\sum }\limits_{j=1}^{M}{\alpha }_{j}^{2}.$$

The Cox Lasso regression estimator ${\hat{\alpha }}^{l}$^27^ can be obtained by minimizing

$$-PL(\alpha )+{\lambda }_{l}^{PH}\mathop{\sum }\limits_{j=1}^{M}|{\alpha }_{j}|.$$

The Cox adaptive Lasso regression estimator ${\hat{\alpha }}^{al}$ can be obtained by minimizing

$$-PL(\alpha )+{\lambda }_{al}^{PH}\mathop{\sum }\limits_{j=1}^{M}\frac{1}{{w}_{j}}|{\alpha }_{j}|,$$

where ${w}_{j}={\hat{\alpha }}_{j}^{l}$ is the jth component of ${\hat{\alpha }}^{l}$.

As noted above, the Cox Lasso and adaptive Lasso regression methods were used for variable selection, and the optimal tuning parameter estimators, ${\hat{\lambda }}_{r}^{PH}$, ${\hat{\lambda }}_{l}^{PH}$ and ${\hat{\lambda }}_{al}^{PH}$, were obtained with the cross-validation method. Similar to the penalized logistic regression methods described above, the Cox Lasso and adaptive Lasso regression methods can be used for variable selection. The genes selected were evaluated based on their association with the survival time distribution.

#### FDR for screening

The method proposed by Benjamini and Hochberg to control the FDR, defined as the expectation of the ratio of the number of falsely rejected null hypotheses to the total number of rejected null hypotheses, was used here^28^. On the App we developed, the method to control the FDR is provided as an optional first-step screening method and users may also specify their own FDR thresholds. In this study, the default FDR threshold was set at 0.05. When FDR was chosen, univariate analysis (Cox regression for a survival outcome and logistic regression for a binary outcome) was first performed to compute the p-value (the extent of the association) for each gene, and then FDR screening was performed. Once the associated genes were selected, the regression model would be fit to the outcome variable with the selected genes as covariates.

#### Cross validation for estimating prediction power

The cross-validation algorithm is provided on the App to estimate the prediction power of each model available on the platform and users are allowed to choose the fold number for the cross validation. The default fold number is 5, and cross validation method will not be performed if 1 is chosen. Accuracy, sensitivity, specificity and area under curve (AUC) are computed and displayed to show the prediction power for each logistic regression model, and the concordance index (C-index) for each survival model.

#### Computing the correct p-values for lasso and adaptive lasso

When running the Lasso (or adaptive Lasso) regression analysis, most statistical software programs do not provide p-values. Computing p-values for the Lasso (or adaptive Lasso) is difficult as both regression methods are involved in the variable selection procedure (see detailed explanation in Lee *et al*.^29^. To solve this problem, Lee *et al*.^29^ developed a general approach to compute the correct p-values after model selection. Here we used the ‘selectiveInference’ R package^29,30^ to implement the algorithm by Lee *et al*. to compute the correct p-values for the Lasso and adaptive Lasso regression.

## Results

### Introduction to HDMAC

**Figure 1:**
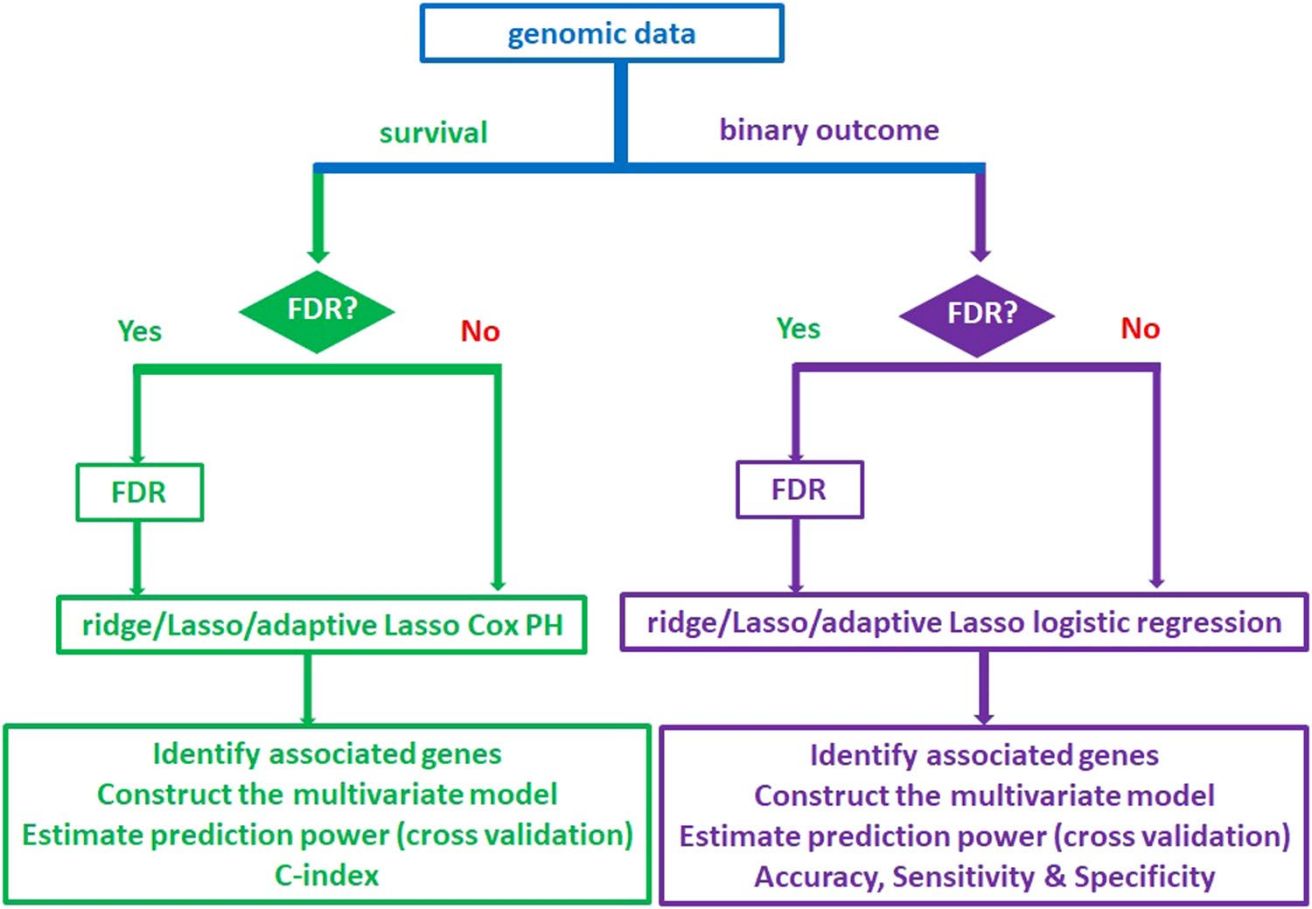
Flowchart of running HDMAC.

We constructed a package of high-dimensional analysis of molecular alterations in cancer, HDMAC, and made it a web-based platform at https://ripsung26.shinyapps.io/rshiny/. A flowchart of running HDMAC is provided in Fig. 1, and the tutorial on how to use it is available both at GitHub (https://github.com/chung-R/HD-MAC) and as a supplementary file (Supplementary Method 1).

On HDMAC, we provided a set of example data for users to get familiar with the platform. For analysis of their own data, users may choose to upload the data and run it through the statistical methods provided. For analysis of data from TCGA, users may take advantage of the R codes we wrote to download whole sets of data from TCGA. These codes help with procurement of large-scale data, and are available at GitHub (see “Data download from TCGA.r” at https://github.com/chung-R/HD-MAC). We have also provided all the codes of the entire platform at GitHub (see folder HDMAC at https://github.com/chung-R/HD-MAC) for researchers to analyze their data offline with RStudio^31^. In addition, all the functions in our App were validated with different R packages and the validation codes were available at GitHub (https://github.com/chung-R/HD-MAC) as well.

Analysis with the statistical methods provided on HDMAC is illustrated in the sections below.

### Survival analysis with serous type high-grade ovarian cancer patients

To show how to analyze survival data, we adopted a set of data of high-grade serous ovarian cancer and ran the data on the HDMAC. The patients’ overall survival was used as the outcome variable.

The three Cox regression methods, the ridge, Lasso and adaptive Lasso, all available on HDMAC, were used to analyze the data in response to overall survival. The ridge regression showed mutations of 670 genes, and each of the Lasso and the adaptive Lasso selected 1 gene.

Then the method to control the FDR was included as the first-step screening. As a result, each of the ridge, Lasso and adaptive Lasso Cox methods selected mutations of 2 same genes, ZSWIM8 and PABPC3.

The above results may be tested for their predictive performance with the cross validation method provided on HDMAC. Here we adopted the 5-fold cross validation to calculate the C-indices of the results above. The C-indices were 0.529, 0.501, and 0.501 for the ridge, Lasso and adaptive Lasso without controlling the FDR, respectively, and with the control for the FDR, the three indices were 0.502, 0.502, and 0.497, respectively.

Similar analysis was then performed on the mRNA expression data. The ridge Cox regression showed 9,548 mRNA expression entries while each of the Lasso and adaptive Lasso selected 4 mRNA entries. Their C-indices were 0.591, 0.554, and 0.560, respectively. When the method to control the FDR was included as the first-step screening, 6 same entries were left to all the three methods, with the respective C-indices being 0.538, 0.538, and 0.540.

All the results above are summarized in Table 1. The 2 mutated genes and the 6 abnormally expressed mRNAs identified with the control for the FDR, as well as their hazard ratios, Table 2.

**Table 1:** Numbers of genes and c-indices with mutations and mRNA expression abnormalities in response to overall survival of ovarian cancer.

Cox PH method		Ridge		Lasso		Adaptive Lasso	
		numbers	c-index	numbers	c-index	numbers	c-index
mutated genes	no FDR	670	0.529	1	0.501	1	0.501
	after FDR	2	0.502	2	0.502	2	0.497
mRNA expression abnormalities	no FDR	9548	0.591	4	0.554	4	0.560
	after FDR	6	0.538	6	0.538	6	0.540

**Table 2:** Genes selected with FDR penalty to be significantly associated with overall survival in ovarian cancer.

Mutated genes	Estimated coefficients	Hazard ratio	p-value	Abnormally expressed genes	Estimated coefficients	Hazard ratio	p-value
ZSWIM8	2.014	7.493	0.00007	ASAP3	0.09	1.094	0.0682
PABPC3	1.729	5.635	0.00071	C10ORF113	0.08	1.083	0.0330
				TIGAR	0.08	1.083	0.0001
				KIAA0100	0.05	1.051	0.0188
				REPL4B	0.007	1.007	0.0036
				ZFHX4	0.08	1.083	0.0231

### Logistic regression analysis on the invasion subtype of bladder cancer

To demonstrate the analysis associated with binary clinical outcomes, we chose a set of bladder cancer data and performed analysis relative to the subtype of bladder cancer, *i.e*., whether or not the patients had invasive or non-invasive tumors. We chose a different set of data to illustrate the analysis with logistic regression here to show that HDMAC was applicable to various types of data. The analysis with a binary outcome based on the ovarian cancer data above and that with survival based on the bladder cancer data here are provided in Supplementary Tables S1 and S2.

As the outcome was binary, we used the ridge, Lasso and adaptive Lasso logistic regression. The ridge logistic regression showed 8,024 mRNA entries, and the Lasso and the adaptive Lasso selected 46 and 27, respectively, in relation to cancer subtype without controlling the FDR. When the method to control the FDR was included, the ridge showed 461 mRNA entries, and the Lasso and the adaptive Lasso, 36 and 24, respectively. We also tested the predictivity of these results by calculating the sensitivity, specificity, accuracy, and AUC based on 5-fold cross validation. All the results above are shown in Table 3. As a relatively large number of genes were selected in each method, we only presented the shortest list, *i.e*., the mRNA entries selected with FDR adaptive Lasso regression, as well as their estimated coefficients in Table 4.

**Table 3:** Numbers of genes and the test statistics of mRNA expression abnormalities in response to the invasion subtype of bladder cancer and the validation results.

Logistic regression	Ridge		Lasso		Adaptive Lasso	
	no FDR	with FDR	no FDR	with FDR	no FDR	with FDR
# abnormal expression	8024	461	46	36	27	24
Sensitivity	0.565	0.500	0.533	0.565	0.484	0.532
Specificity	0.701	0.764	0.709	0.677	0.772	0.717
Accuracy	0.656	0.677	0.651	0.640	0.677	0.656
AUC (area under curve)	68.107	66.515	65.864	67.020	62.442	64.300

**Table 4:** Genes selected with adaptive Lasso logistic regression after FDR penalty whose abnormal expression was associated with invasion in bladder cancer together with their estimated coefficients, odds ratios and p values.

Abnormally expressed genes	Estimated coefficients	Odds ratio (ln)	p-value
SPTSSA	−0.16	0.852	0.51
ATAT1	0.06	1.061	0.47
CABP4	0.26	1.296	0.11
CCNK	−0.27	1.309	0.19
CIR1	0.55	1.733	0.50
DPP9	0.42	1.521	0.05
FANCL	0.01	1.010	0.92
ICOSLG	−0.66	0.516	0.004
JOSD1	−0.35	0.704	0.54
MED30	−0.43	0.650	0.01
NADSYN1	−0.71	0.491	0.27
NCOA3	−0.52	0.594	0.003
LINC00173	−0.12	0.886	0.66
NKIRAS1	−0.29	0.748	0.10
NUDT16P1	0.24	1.271	0.15
PDRG1	−0.69	0.501	0.49
POLR1D	0.55	1.733	0.02
PSORS1C2	1.14	3.126	0.005
RETSAT	−0.32	0.726	0.18
RPL23AP7	0.66	1.934	0.01
SETMAR	0.29	1.336	0.52
SLC14A1	0.50	1.648	0.05
SLC39A4	0.14	1.150	0.65
ZSCAN2	0.27	1.309	0.16

### Multivariate model building

Once genes are selected with their corresponding coefficients, a multivariate model may be built. For example, the coefficients of the abnormally expressed genes found to be associated with the invasive subtype of bladder cancer with the adaptive Lasso regression after the FDR penalty, as listed in Table 4, may be used to construct a multivariate model as follows:

$$\log (\frac{\hat{{\rm{P}}}({Y}_{i}=1|{X}_{i})}{\hat{{\rm{P}}}({Y}_{i}=0|{X}_{i})})=-\,0.16\,\times \,{\rm{SPTSSA}}+0.06\,\times \,{\rm{ATAT}}1+\ldots +0.14\,\times \,{\rm{SLC}}39{\rm{A}}4+0.27\,\times \,{\rm{ZSCAN}}2$$

A positive coefficient indicates that the gene’s abnormal expression is positively associated with the invasive subtype while a negative one, negatively. The result of the above function could be used to predict whether a patient has invasive bladder cancer with a given threshold. In this study, the threshold was set at 0.34 such that a patient with a score calculated from the above function higher than 0.34 would be predicted to have the invasive subtype of bladder cancer and vice versa.

## Computation time

Since the data to be analyzed on HDMAC may be extremely big with a large number of observations and/or a large number of variables, there may be concerns about how efficient HDMAC is. We thus tested the computing time and uploading time with simulation of different situations of observations/numbers. Tables 5 and 6 show the uploading time and the average computing time, respectivly, for both logistic and survival analyses. Each table shows the results of 9 combinations with a small (50), a medium (200) and a large (1000) number of observations and a small (50), a medium (500) and a large (5000) number of variables. All the analyses for the simulation were performed using the online version of HDMAC. The simulated data were generated based on the real datasets we used in this paper. The simulation was conducted using the adaptive Lasso and Lasso for logistic regression analysis and survival analysis, respectively, to keep consistency with the real data analysis.

**Table 5:** Uploading time for both logistic regression and survival analysis (seconds).

Number of Observations	Number of variables		
	Small (50)	Medium (500)	Large (5000)
Small (50)	1.1	1.8	5.1
Medium (200)	1.5	3.5	12.4
Large (1000)	3.4	8.4	54.9

**Table 6:** Computing time for logistic regression and survival analysis (seconds).

Number of Observations	Number of variables					
	Logistic regression			Survival analysis		
	Small (50)	Medium (500)	Large (5000)	Small (50)	Medium (500)	Large (5000)
Small (50)	1.5	1.7	4.5	1.4	1.6	2.5
Medium (200)	1.7	1.9	5.5	1.6	5.8	14.1
Large (1000)	4.3	6.4	16.4	12.8	59.2	128.2

As expected, with the increasing numbers of observations and variables, the computing time for the survival analysis and that for the logistic regression analysis increased. As the numbers of observations and variables increased, the uploading time also increased. When the number of observations was large, the computing time for the survival analysis increased much more than that for the logistic regression analysis. In addition, as the numbers of observations and variables were both very large, the uploading time increased significantly.

## Discussion

Cancer has become one of the top killers in the present world^32^. Recent advances in high-throughput assays and genomic analysis have greatly enriched our understanding of genetic alterations underlying the etiology of cancer. However, there is a growing need for convenient use of solid and rigorous statistical tools, especially those that are able to address the high dimensionality of genomic data. HDMAC, the platform we developed, has the following advantages. It provides regularized regression to analyze high-dimensional data and is the only web-based software that offers penalized Cox regression for survival analysis. For logistic regression, HDMAC offers the adaptive Lasso regression, which is important for variable selection but rarely found in other web-based tools. It also provides users with many statistical analyses in one single platform, including the first step screening (FDR method) and p-value corrections that usually require users to download specific packages or even navigate to a different platform. Furthermore, HDMAC is web based and no code writing or downloading is needed.

HDMAC is a user-friendly, interactive and web-based platform. Few such platforms for genetic analysis have been developed in the literature, among which the GEPIA and UALCAN are closest to our purpose. While both GEPIA and UALCAN are useful web-based interactive tools to analyze cancer OMICS data and suitable for exploratory analysis and visualization, the most important advantage of HDMAC is that it includes high dimensional regression analysis, and the other two do not. Here high-dimensional regression analysis is to analyze how thousands of or even more, hence high-dimensional, variables affect the outcome at the same time. It is not univariate analysis for many variables which many web-based platforms for omics data analysis do (*i.e*., many genes are considered, but each analysis only involves one gene), or traditional multivariate regression analysis which only deals with at the most dozens of variables each time. The purpose of the high-dimensional regression analysis using HDMAC is to explore the effect of the “high-dimensional” genetic variables combined on the outcome, select important variables and estimate their prediction power for the outcome. As far as we know, HDMAC is the only web-based interactive tool that offers high-dimensional regression analysis although such analysis has been used intensively for OMICS data. Moreover, GEPIA and UALCAN only have univariate survival analysis, and HDMAC offers both survival and logistic regression analyses, with both univariate and multivariate options. Furthermore, HDMAC can analyze many kinds of OMICS data such as gene expression, copy number variation, mutation, protein expression, methylation, etc., while the other two platforms are more focused on specific OMICS data such as gene expression on GEPIA and gene expression and methylation on UALCAN.

There are other apps that are related to HDMAC, e.g., CASAS is a web-based app for survival analysis and MLJAR (at https://mljar.com/) is a web-based tool for logistic regression analysis. However, CASAS offers only univariate Cox regression analysis for one or several user-specified variables, but not for high dimensional penalized Cox regression analysis^12^, and MLJAR is for traditional, not regularized, logistic regression. There are several apps that provide some penalized regression analysis that are also available on HDMAC. Compared to these apps, HDMAC has the advantage of offering these functions readily without any need to write codes or download additional packages. For example, both Tensorboard and Weka require users to download and install software and/or packages or even write codes to run the regularized logistic regression although only Lasso and Ridge, and not adaptive lasso, regression can be downloaded^22–24^. Similarly, for first step screening or conducting significance test for the Lasso and adaptive Lasso regression, currently available apps require users to either download other packages or to run them using other apps.

For more specific functions for statistical inference, HDMAC provides validation methods for prediction power so that researchers will be aware of how much confidence they may have in their results. Therefore, if a higher prediction power is desired, users may rely on the validation test, e.g., C-index for a survival outcome and accuracy for a binary one, for the final choice of a regression method. In contrast, if variable selection is preferred, the Lasso and the adaptive Lasso are best choices. In particular, HDMAC offers an algorithm to calculate the correct p values for the Lasso and adaptive Lasso methods, which are not usually available in common statistical software due to the methods’ involvement in variable selection. In addition to the statistical strength mentioned above, we also provided a method to control the FDR as the first-step screening. It is an optional choice for users to address the multiple-testing problem that arises when they study the associations among many molecular variables at the same time. Inclusion of FDR is recommended if users are dealing with variables at the magnitude of a hundred thousand where penalized regression models fall short. In addition, clinical variables such as gender and age may also be included in the analysis although they were not illustrated in the results above.

We have provided on GitHub both the R scripts of HDMAC that enable Rstudio users to use all the analysis on HDMAC offline and the R script to download data from the TCGA. Meanwhile, it is worth noting that users can use HDMAC with any data while the TCGA database is just one important source. Also, there are several existing useful tools to download the TCGA data in addition to the R script we provided. For example, FireBrowse portal allows for downloading TCGA data directly through a web UI (Firebrowse.org), and TCGAbiolinks (https://bioconductor.org/packages/release/bioc/html/TCGAbiolinks.html) is also a useful R package to this end. Compared to TCGAbiolinks, our R script has the advantage that it was written with a hierarchical structure where users are guided step-by step to download a TCGA dataset. At each step, users can see the options they have on the screen and immediately know the key words they need to enter at the next step.

Ovarian cancer, especially the serous type high-grade ovarian cancer, is a major threat to women. It is the seventh most common cancer among women, but the second leading cause of gynecologic cancers worldwide, with estimated 295,414 new cases and 184,799 deaths in 2018^32^. Most women are diagnosed with ovarian cancer at an advanced stage, and the overall 5-year survival rate ranges between 30% and 40%, which has seen only extremely modest improvement since 1995^33^.

Some molecular changes are known to predispose the development of ovarian cancer. The most studied genes are *BRCA1* and *BRCA2*^34–36^. Other genes, such as *CHEK2*, *ATM*, and *PALB2* and Lynch syndrome genes, are also implicated in ovarian cancer^37^. Overall, however, genome-wide search for genetic changes associated with survival in ovarian cancer is still waiting. Our efforts in this study came up with a preliminary list of genes worth further study in depth, such as ASAP3 [26886260].

Bladder cancer is the most common cancer of the urinary tract and the ninth most common cancer worldwide, with estimated 549,393 new cases and 199,992 deaths in 2018^32^. Its incidence is observed to be strongly prevalent in males, with approximately a men-to-women ratio of 3:1, and it is strongly associated with smoking^38^. Approximately 80% of newly diagnosed patients are identified as the non-muscle invasive subtype (NMIBC; stages Ta/T1), while the remaining 20% are muscle invasive (MIBC; stages T2-4)^39^. Due to distinct cancerous behaviors and clinical outcome, their respective origins remain controversial^40–42^. Therefore, it is highly desirable to explore molecules involved in the interplay and transition between these two subtypes.

A variety of chromosomal alterations, including mutations, copy number changes and allelic losses, in combinations of multiple genetic signatures, have been linked to bladder cancer such as changes in *FGFR3*, activation of cellular signaling in PI3K, MAPK and WNT pathways, or dysregulation of genes involved in cell cycle^43^. However, whether those alterations drive bladder cancer to become more aggressive needs further investigation. The genes identified in this study, although still preliminary, provide rational directions to further explore molecular links that control the switch for transition between the two types. Notably, different lines of evidence have already suggested the usefulness of our predicted gene candidates. For examples, genetic variations in *SLC14A1* have been linked to the development of bladder cancer^44,45^ and its upregulation has been suggested as a potential target for clinical intervention^46,47^. In addition, a negative regulatory role of MED30 has been recently revealed in that its overexpression can suppress the progression of bladder cancer^48^.

In summary, the HDMAC platform we developed offers a solution for rigorous analysis of high-dimensional genomic data. It is clinically oriented and user friendly while including statistical methods to address major issues in large-scale data analysis. It thus has a potentially wide application.

## Supplementary information

Supplementary Information.
