# Supplementary material for: HDMAC: A Web-Based Interactive Program for High-Dimensional Analysis of Molecular Alterations in Cancer
Source: Sci Rep. 2020 Mar 3;10:3953. doi: 10.1038/s41598-020-60791-z (PMC7054321; doi:10.1038/s41598-020-60791-z)
Supplement: Supplementary file 1 — Supplementary Information. [file 41598_2020_60791_MOESM1_ESM.pdf]

HDMAC: A Web-Based Interactive Program for High-Dimensional Analysis of Molecular Alterations in Cancer

Chung Chang[1], Chan-Yu Sung[1], Han Hsiao[1], Jiabin Chen[2], I-Hsuan Chen[3-5], Wei-Ting Kuo[3], Lung Fung Cheng[3], Praveen Kumar Korla[2], Ming-Jhe Chung[1], Pei-Jhen Wu[1], Chia-Cheng Yu[3-5, 6, *], Jim Jinn-Chyuan Sheu[2, 7-9, *]

1, Department of Applied Mathematics, National Sun Yat-sen University, Taiwan, ROC

2, Institute of Biomedical Science, National Sun Yat-sen University, Taiwan, ROC

3, Department of Surgery, Kaohsiung Veterans General Hospital, Kaohsiung 81362, Taiwan, ROC

4, Department of Pharmacy, College of Pharmacy and Health Care, Tajen University, Pingtung County 90741, Taiwan, ROC

5, School of Medicine, National Yang-Ming University, Taipei 112, Taiwan, ROC

6, Department of Surgery, Tri-Service General Hospital, National Defense Medical Center, Taipei 114, Taiwan, ROC

7, Human Genetic Center, China Medical University Hospital, Taichung 40447, Taiwan, ROC

8 School of Chinese Medicine, China Medical University, Taichung 40402, Taiwan, ROC

9 Department of Health and Nutrition Biotechnology, Asia University, Taichung 41354, Taiwan, ROC

*Corresponding Authors
Primary correspondence to Jim Jinn-Chyuan Sheu at
National Sun Yat-sen Univeristy
70 Lienhai Road, Kaohsiung 804, Taiwan, R.O.C.
Tel: 886-7-5252000 Fax: 886-7-5253809
Email: jimsheu@mail.nsysu.edu.tw

HDMAC Tutorial

The high-dimensional analysis of cancer-associated genetic alterations, HDMAC, was developed to analyze high-dimensional genetic covariates, with the choice of including clinical covariates, with several regression models suitable for high-dimensional analysis and to identify important genetic alterations which could be used to construct a fitted multivariate regression model while its prediction power could be estimated by cross validation. HDMAC also allows choice of a penalty type for the corresponding penalized regression model for high-dimensional data and a first-step screening to screen out unrelated variables if the multiple-testing problem is of concern via control of the false discovery rate (FDR). Below is a tutorial on how to use HDMAC with respect of both survival data and binary outcome. The platform is available at http://ripsung26.shinyapps.io/rshiny.

Data Upload/Toy Example

To begin the analysis of your data, go to the website listed above and click the tab “Upload Data”. On the left of the page, you may choose whether to upload your own data or use the toy example we provided on the site. The data you upload or from our toy example are shown on the right. You may also choose which columns are shown on the front page.

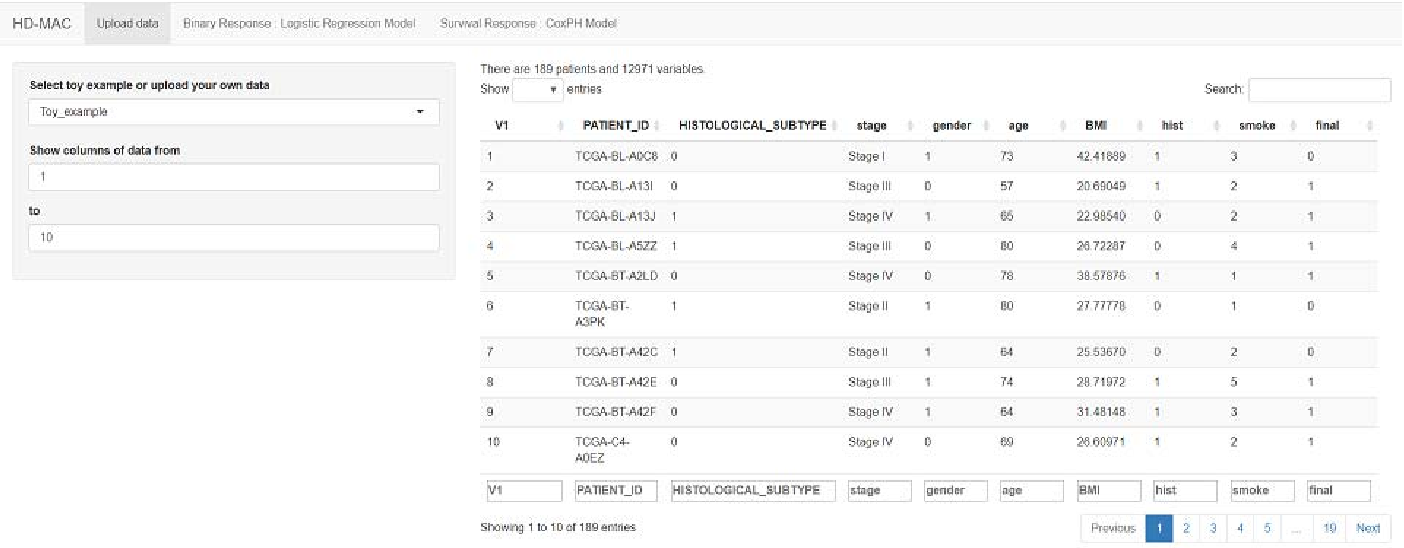

V1	PATIENT_ID	HISTOLOGICAL_SUBTYPE	stage	gender	age	BMI	hist	smoke	final
1	TCGA-BL-A0C8	0	Stage I	1	73	42.41889	1	3	0
2	TCGA-BL-A13I	0	Stage III	0	57	20.68049	1	2	1
3	TCGA-BL-A13J	1	Stage IV	1	85	22.98540	0	2	1
4	TCGA-BL-A5ZZ	1	Stage III	0	80	26.72287	0	4	1
5	TCGA-BT-A2LD	0	Stage IV	0	78	38.57876	1	1	1
6	TCGA-BT-A3PK	1	Stage II	1	80	27.77778	0	1	0
7	TCGA-BT-A42C	1	Stage II	1	64	25.53670	0	2	0
8	TCGA-BT-A42E	0	Stage III	1	74	28.71972	1	5	1
9	TCGA-BT-A42F	0	Stage IV	1	64	31.48148	1	3	1
10	TCGA-C4-A0EZ	0	Stage IV	0	69	26.60971	1	2	1

The size limit to the data you upload is 50 MB. It may take a few moments for your data to upload dependent on the size.

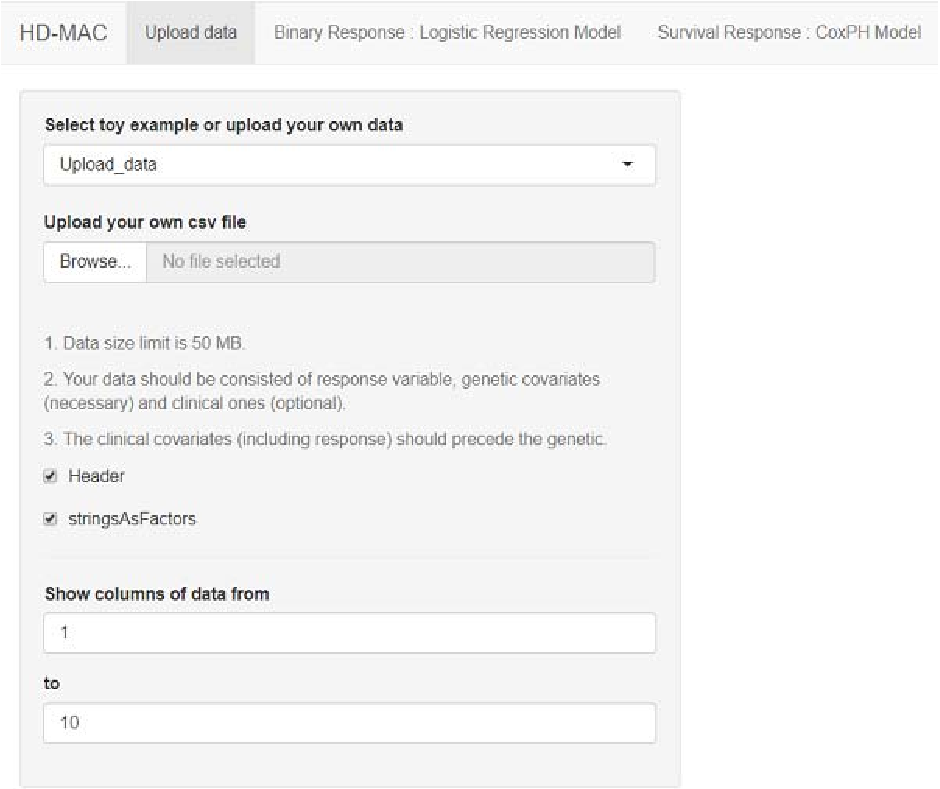

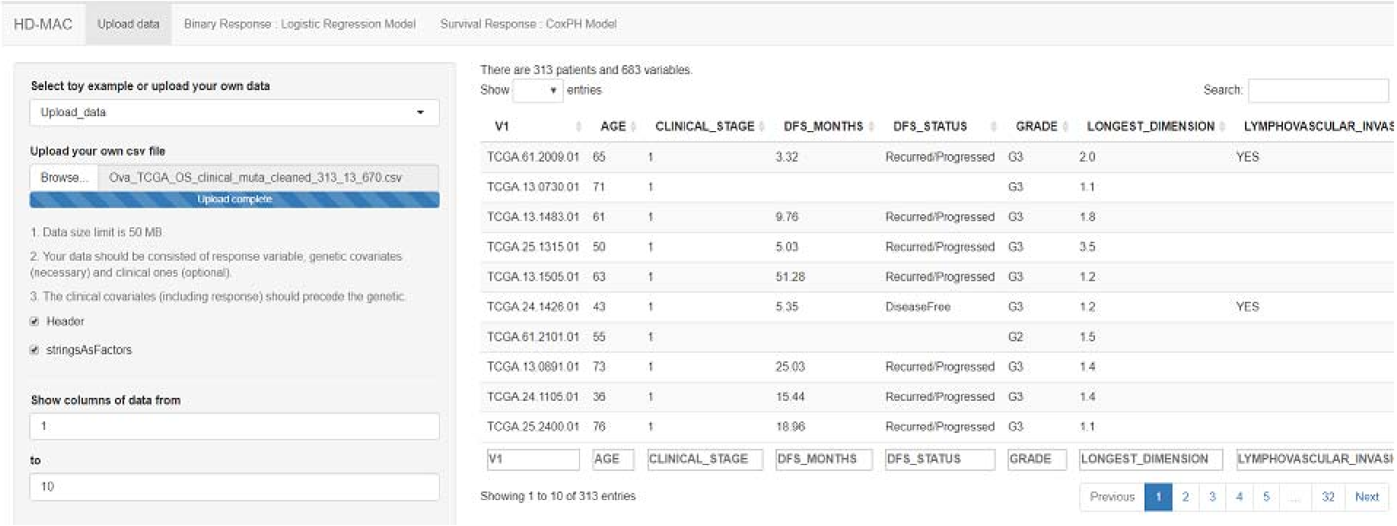

Once the data are uploaded, you may begin your analysis. For survival data, click the tab “Survival Regress: CoxPH Model”. For binary outcomes, click the tab “Binary Regression: Logistic Regression Model”.

Survival Analysis

The data used here to illustrate how to run survival analysis on HDMAC contain the information of 8,310 mutated genes from 316 patients with serous type, high grade ovarian cancer from TCGA. The aim is to relate gene mutations to the patients’ overall survival.

1. Choose the tab “Survival Response: CoxPH Model” and locate all the variables needed for the analysis in the data uploaded. Inclusion of clinical variables is optional. Then choose the Cox regression method desired. Three are available: ridge, Lasso and adaptive Lasso.

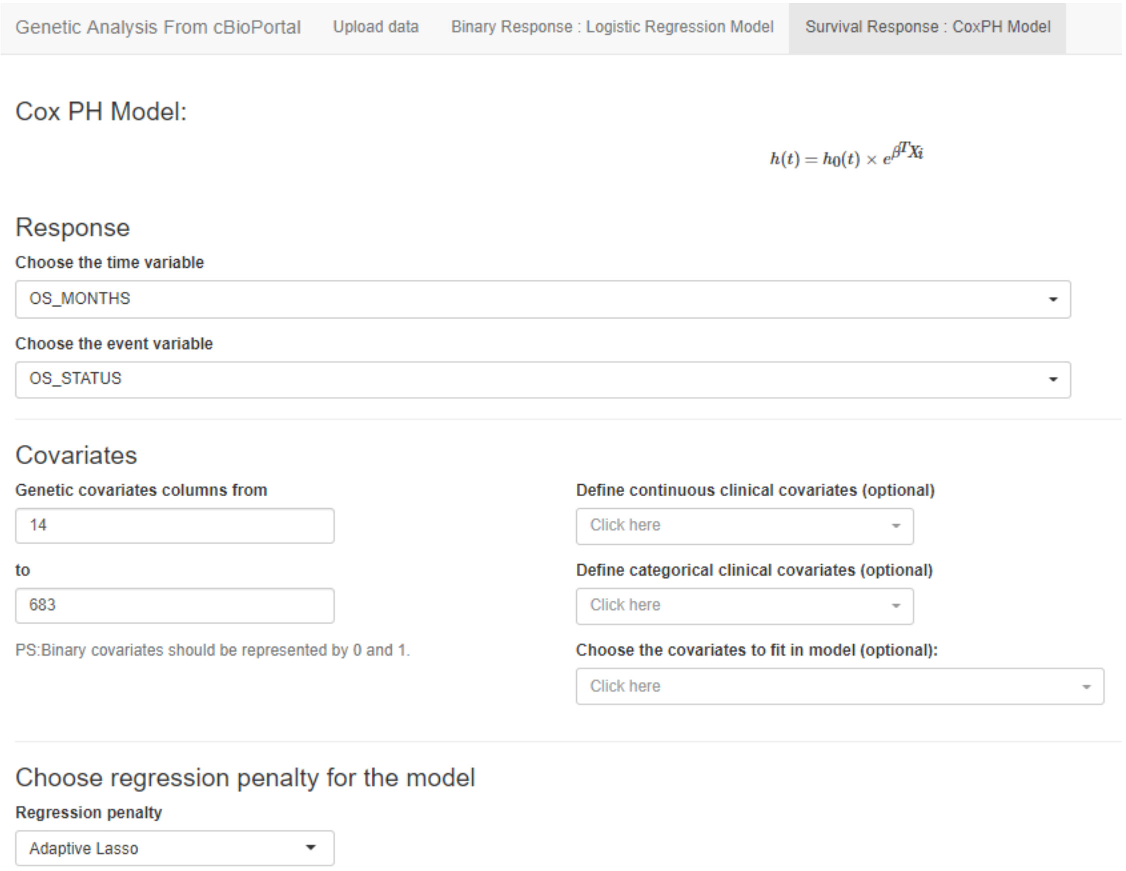

2. Print the gene list. [Optional:] Choose whether the initial screening to control the FDR is desired. If chosen (the box before "Use FDR for screening" is checked as shown below), the p-value threshold is set at 0.05. The number of cross-validation (CV) folds for testing the prediction power (C-index in the case of survival analysis) is set at 1 as the default for printing out gene lists. It is possible to change the CV fold for statistical tests, which is illustrated in the next step.

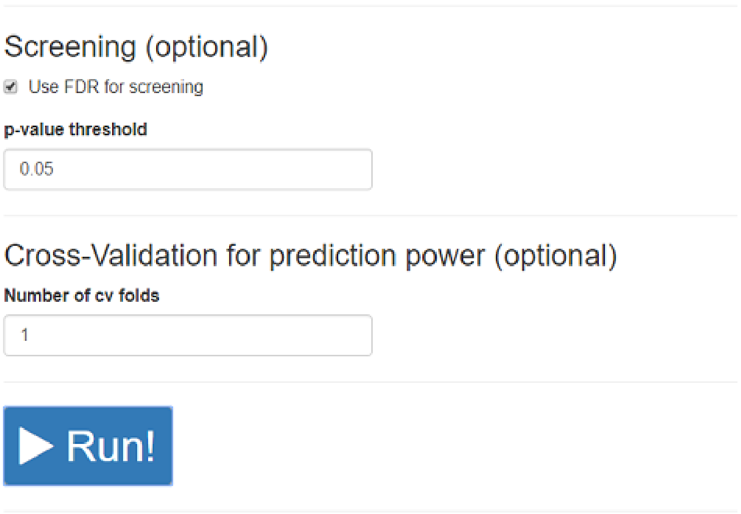

Hit "Run" and the gene list with each gene's coefficient and p value will be printed on the page.

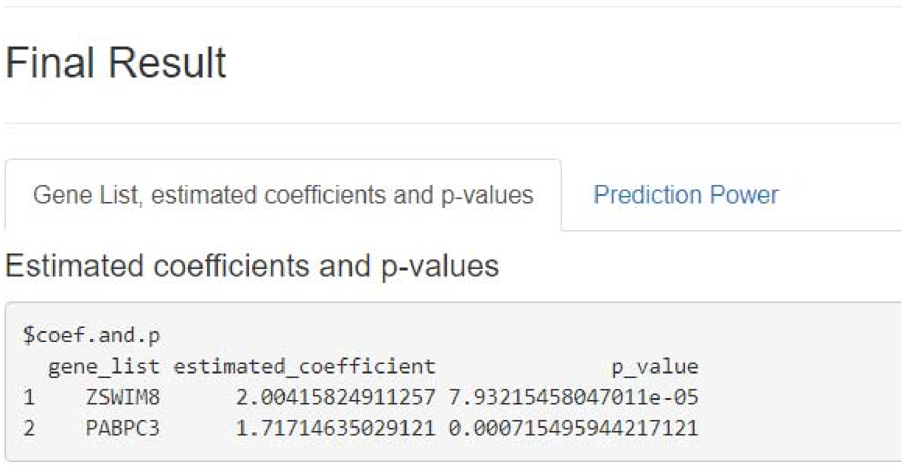

3. (Optional) To test the prediction power of the results, set the CV folds at 5 and hit RUN.

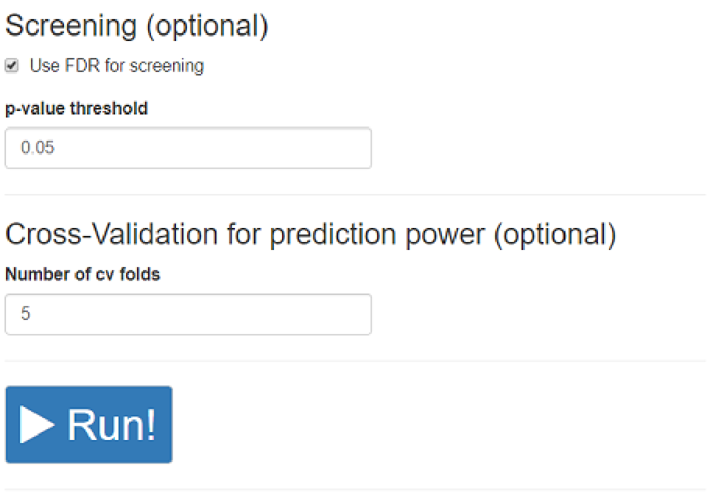

The concordance index, C-index, will be calculated to show the prediction power.

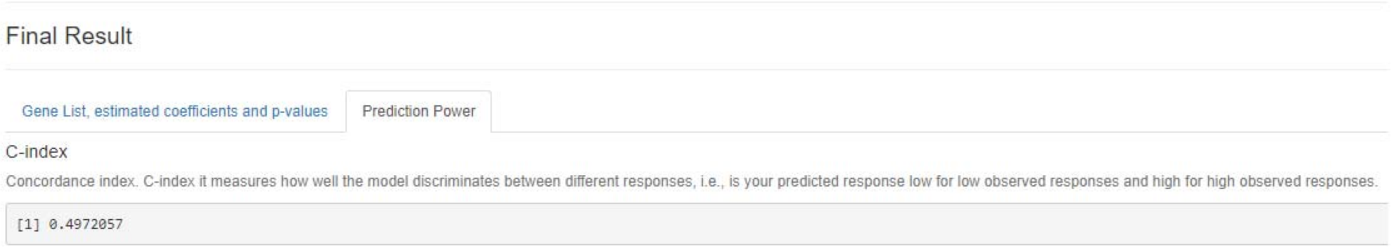

<u>Binary Outcome</u>

The data used here to illustrate how to run logistic regression in response to a binary outcome on HDMAC contain the information of 18,335 entries of mRNA expression of 189 patients with bladder cancer from TCGA. The aim is to relate abnormal mRNA expression to the patients' cancer subtype, invasive vs. non-invasive.

1. Choose the tab “Binary Response: Logistic Regression Model” and locate all the variables needed for the analysis in the data uploaded. Inclusion of clinical variables is optional. Then choose the logistic regression method desired. Three are available: ridge, Lasso and adaptive Lasso.

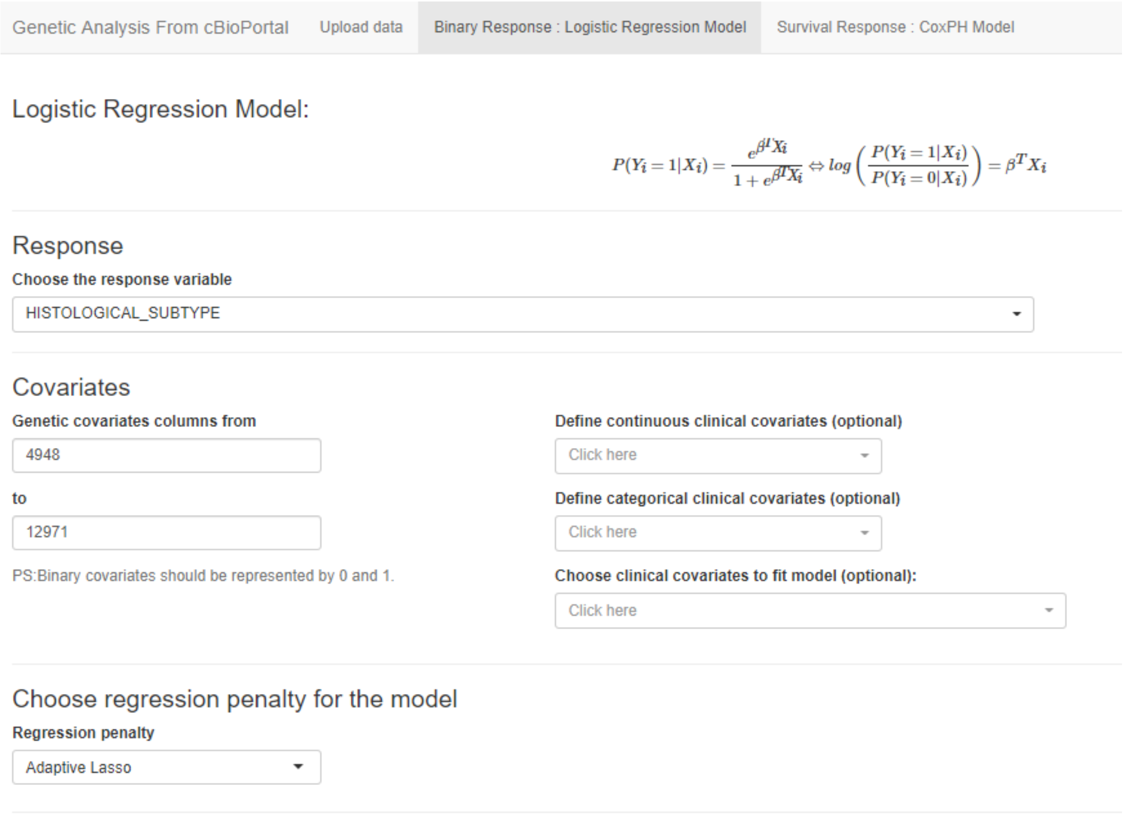

2. Print the gene list. [Optional:] Choose whether the initial screening to control the FDR is desired. If chosen (the box before “Use FDR for screening” is checked as shown below), the p-value threshold is set at 0.05. The number of cross-validation (CV) folds for testing the prediction power (sensitivity, specificity, accuracy and area under curve (AUC) in the case of logistic regression) is set at 1 as the default for printing out gene lists. It is possible to change the CV fold for statistical tests, which is illustrated in the next step.

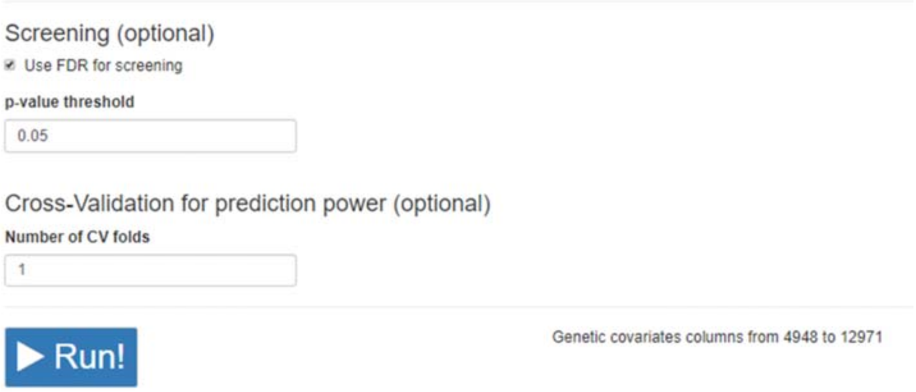

Hit “Run” and the gene list with each gene’s coefficient and p value will be printed on the page.

Final Result

Gene List, estimated coefficients and p-values | Prediction Power

Gene list, estimated coefficients and p-values

```
$coef.and.p
   gene_list estimated_coefficient              p_value
1     SPTSSA       -0.156997435976    0.507432675387873
2      ATAT1    0.0645544675302372    0.465140453927364
3      CABP4      0.25563910739168    0.105231535604245
4       CCNK    -0.267889957896636    0.189086746946374
5       CIR1     0.548111051382859    0.498533011172238
6       DPP9     0.417290082265505   0.0513867305149367
7      FANCL   0.00811012698940946    0.921298999547267
8     ICOSLG    -0.661599439029748  0.00457770627109811
9      JOSD1     -0.34525522387081    0.536529827233434
10     MED30    -0.427390776433774  0.00509724460953874
11   NADSYN1     -0.71086241148283    0.267423965046945
12     NCOA3    -0.522909533503091  0.00328788258680913
13 LINC00173    -0.122313676831379    0.657342971141993
14   NKIRAS1    -0.291523233294147   0.0980830089761888
15  NUDT16P1     0.243261116801207    0.154617778676624
16     PDRG1    -0.693371786736194    0.485094004547082
17    POLR1D     0.548242355818826   0.0163494427594439
18  PSORS1C2        1.141490537781 8.37984637017003e-05
19    RETSAT    -0.316509356633728    0.179141161077715
20  RPL23AP7    -0.656214021472041  0.00865333695527046
21    SETMAR      0.28719381446673    0.519002797134722
22   SLC14A1     0.502245058310408   0.0529791613094708
23   SLC39A4     0.138888570580463    0.653141097318773
24    ZSCAN2      0.27041666667045    0.161834300293155
```

3. (Optional) To test the prediction power of the results, set the CV folds at 5 and hit RUN.

Screening (optional)

- [x] Use FDR for screening

p-value threshold

0.05

Cross-Validation for prediction power (optional)

Number of CV folds

5

▶ Run!

Genetic covariates columns from 4948 to 12971

The sensitivity, specificity, accuracy, and AUC will be calculated to show the prediction power.

Final Result

Gene List, estimated coefficients and p-value | Prediction Power

Sensitivity

```
[1] 0.516129
```

Specificity

```
[1] 0.6771654
```

Accuracy

```
[1] 0.6243386
```

AUC (%)

```
[1] 0.635588
```

Supplementary Table S1. Logistic regression methods in analysis on mRNA expression abnormalities and gene mutations in response to lymphovascular invasion of ovarian cancer and validation

Logistic regression		Ridge		Lasso		Adaptive Lasso	
		mRNA	mutation	mRNA	mutation	mRNA	mutation
number of genes		9548	567	28	5	17	5
Test statistics	Sensitivity	0.750	0.609	0.643	0.913	0.667	0.913
	Specificity	0.581	0.476	0.395	0.048	0.465	0.048
	Accuracy	0.693	0.559	0.559	0.586	0.598	0.586
	AUC *	68.294	52.322	62.103	47.598	63.104	47.598

*AUC, area under curve.

Genes selected by the adaptive Lasso

Mutated genes	Estimated coefficients	log odds	p-value	Abnormally expressed genes	Estimated coefficients	log odds	p-value
ANKRD11	-0.0993	0.9054	0.7050	CDR2L	0.42	1.521	0.12
BPIFB2	-0.1331	0.8753	0.7417	CTSD	0.29	1.336	0.43
GAB2	-0.0992	0.9055	0.7997	HNRNPAB	-0.77	0.463	0.00
IDSF10	-0.0997	0.9051	0.7033	UFL1	-0.32	0.726	0.10
VSIG2	-0.0997	0.9051	0.7050	LONP2	-0.69	0.501	0.00
				PCNP	-0.18	0.835	0.23
				RFXAP	-0.13	0.878	0.40
				SALL2	-0.15	0.860	0.20
				SCAMP2	0.25	1.284	0.19
				SPINK5	-0.07	0.932	0.64
				ZNF74	-0.06	0.941	0.69

Supplementary Table S2. Numbers of genes and c-indices with mutations and mRNA expression abnormalities in response to overall survival of bladder cancer

Cox PH method		Ridge		Lasso		Adaptive Lasso	
		numbers	c-index	numbers	c-index	numbers	c-index
mutated genes	no FDR	4937	0.566	2	0.506	2	0.506
	after FDR	28	0.468	13	0.484	11	0.495
mRNA expression abnormalities	no FDR	8024	0.547	10	0.595	10	0.576
	after FDR	6	0.586	6	0.603	5	0.609

Genes selected by the adaptive Lasso

Mutated genes	Estimated coefficients	Hazard ratio	p-value	Abnormally expressed genes	Estimated coefficients	Hazard ratio	p-value
BCAS3	1.07	2.915	0.12	EFCAB1	0.16	1.521	0.02
C2ORF42	2.27	9.679	0.02	NEBL	0.26	1.296	0.02
YAE1D1	0.83	2.293	0.42	RASAL2	0.15	1.161	0.01
CNN1	1.29	3.632	0.29	SLC1A6	0.34	1.404	0.00
DNAJB11	1.78	5.929	0.08	UCHL5	0.05	1.051	0.24
IFNGR2	3.50	33.11	0.00				
MKL2	0.89	2.435	0.23				
NRXN3	1.70	5.473	0.02				
NUB1	2.66	14.29	0.00				
OR4A47	2.40	11.02	0.01				
TROAP	0.22	1.246	0.83				
